# Japanese encephalitis virus replicon-based vaccine expressing enterovirus-71 epitope confers dual protection from lethal challenges

**DOI:** 10.1186/s12929-015-0181-8

**Published:** 2015-09-11

**Authors:** Yi-Ting Huang, Jia-Teh Liao, Li-Chen Yen, Yung-Kun Chang, Yi-Ling Lin, Ching-Len Liao

**Affiliations:** Graduate Institute of Life Sciences, National Defense Medical Center, No. 161 Section 6, Ming Chuan E. Road, Taipei, 114 Taiwan Republic of China (ROC); Department of Microbiology and Immunology, National Defense Medical Center, No. 161 Section 6, Ming Chuan E. Road, Taipei, 114 Taiwan ROC; Department of Biochemistry, National Defense Medical Center, No. 161 Section 6, Ming Chuan E. Road, Taipei, 114 Taiwan ROC; Institute of Biomedical Sciences, Academia Sinica, No. 128 Section 2, Academia Road Nankang, Taipei, 115 Taiwan ROC; National Institute of Infectious Diseases and Vaccinology, National Health Research Institutes, No. 35, Keyan Road, Zhunan, Miaoli County, 35053 Taiwan ROC

**Keywords:** Japanese encephalitis virus (JEV), Replicons, Single-round infectious particles (SRIPs), RNA vaccine, Enterovirus-71 (EV71), Neutralizing epitope, SP70, Dual protective immunity

## Abstract

**Background:**

To construct safer recombinant flavivirus vaccine, we exploited Japanese encephalitis virus (JEV) replicon-based platform to generate single-round infectious particles (SRIPs) that expressed heterologous neutralizing epitope SP70 derived from enterovirus-71 (EV71). Such pseudo-infectious virus particles, named SRIP-SP70, although are not genuine viable viruses, closely mimic live virus infection to elicit immune responses within one round of viral life cycle.

**Results:**

We found that, besides gaining of full protection to thwart JEV lethal challenge, female outbred ICR mice, when were immunized with SRIP-SP70 by prime-boost protocol, could not only induce SP70-specific and IgG2a predominant antibodies but also provide their newborns certain degree of protection against EV71 lethal challenge.

**Conclusions:**

Our results therefore exemplify that this vaccination strategy could indeed confer an immunized host a dual protective immunity against subsequent lethal challenge from JEV or EV71.

## Background

Using whole virus particles, conventional live-attenuated or inactivated vaccines have succeeded effective control of several viral infections [1]. To improve vaccine safety and availability, nucleic acid vaccines, including DNA, RNA and engineered viral vectors have emerged as the next generation vaccine strategy for they can effectively mimic vaccination with a live-attenuated virus but without its potential complications [2]. As RNA viral vectors are deliberately engineered to contain subgenomic replicons lacking viral structural protein genes, single-round infectious particles (SRIPs) can be therefore produced in the presence of viral structural proteins that are provided *in trans* by packaging cell line. Those self-replicating RNA replicons were mostly derived from positive-sense RNA viruses, such as Semliki Forest virus, Sindbis virus, poliovirus, tick borne encephalitis virus (TBE), Kunjin virus, and bovine viral diarrhea [2]. The technology of SRIP generation facilitates a rapid and simple engineering of recombinant antigen expression without handling infectious progenies, which appears to be the essence for the next generation vaccine development.

Japanese encephalitis virus (JEV) belongs to a family *Flaviviridae* genus *Flavivirus*, *which* is an enveloped, positive sense, single-stranded RNA virus with an 11-kb genome that encodes three structural proteins of capsid (C), pre-membrane (prM) and envelope (E) and seven non-structural proteins of NS1 to NS5. Of them, prM, E and NS1 proteins could confer the immunized mice a protective immunity against lethal JVE challenge [3]. NS1 is not only expressed on the surface of the infected cells but also secreted outside from infected cells [4]. Because of its high immunogenic nature, NS1 alone is sufficient to trigger protective immunity against subsequently lethal JEV challenges [5].

JEV SRIPs have been shown to be protective against homologous lethal challenges for newborns that were born to the immunized dams [6]. Chimeric flavivirus SRIPs of TBE and JEV that included heterologous antigens were previously established [7–9]. SRIP vaccines seem to be capable of inducing better immune responses than their inactive vaccine counterparts, and more importantly, SRIP vaccines appear to be safer than live attenuated counterparts [6, 10]. Characteristically, JEV vaccines, both live attenuated and inactivated forms, are highly effective and safe among several flavivirus vaccines that currently are routinely used by many Asian and South Asian Counties. It is therefore tempting to develop dual/multiple protective vaccines using JEV genome as a backbone for insertion and expression of heterologous epitopes of interest.

Enterovirus 71 (EV71) together with JEV are the major viral pathogens that attack the central nervous system (CNS) and may cause severe encephalitis in children and young adults in many Asian Countries [11, 12]. Neutralizing epitopes, SP55 (amino acids 163–177) and SP70 (amino acids 208–222) derived from structural protein VP1, have been identified to elicit protective immunity against lethal EV71 challenge in neonatal mice from their immunized mothers [13]. SP70 appears to be especially conserved among various sub-genomic groups of different EV71 strains [13, 14]. We in this study exploited JEV replicon-based platform to generate SRIPs, which were designed to express NS1-fusion proteins containing heterologous neutralizing epitope SP70 from EV71. Such pseudo-type virus particles even though are not genuine viable viruses, can imitate a live virus infection to elicit immune responses from the given hosts within one round of viral life cycle. The vaccine strategy of this kind hence provides a dual protective immunity resulting from a mono vaccine immunization protocol.

## Methods

### Viruses and cells

JEV was recovered from BHK-21 cells transfected with RP-9 infectious cDNA clone (rRP-9) [15] and then was propagated in mosquito C6/36 cells cultured by RPMI 1640 medium containing 5 % fetal bovine serum (FBS, GIBCO). MP4 was a mouse-adapted EV71 strain and grown in Rhabdomyosarcoma (RD) cells in complete Dulbecco’s modified Eagle’s medium/high glucose (DMEM/HG, GIBCO) containing 10 % FBS and 2 mM L-glutamine (GIBCO) [16], which was a kind gift from Dr. Shin-Ru Shih at Chang Gung University, Taiwan. African green monkey kidney (Vero) cells (a kind gift from ADIMMUNE Cor., Taiwan) were grown in MEM with Earle’s Balanced Salt Solution (MEM/EBSS, GIBCO) supplemented with 10 % FBS and 2 mM L-glutamine.

### Construction of JEV DNA-based replicons

To generate JRP and JRPSP70, the genes of Renilla luciferase (RLuc) and 2A-protease were deleted from previously reported replicons of J-R2A [17]. SP70 epitope was fused with NS1 at its C-terminus using Age1 and Kpn1 and R2ASP70 resulted. Nucleotides from 198–2387 corresponding to most of the structural proteins *CprME* were deleted from all JEV replicons used in this study, which also retained capsid-coding sequences from nucleotides 1–197 for genomic RNA cyclization during replication and E-coding sequences from nucleotides 2388–2477 to be a signal sequence for NS1.

### Packaging cells for SRIPs

The PCR fragment of JEV CprME genes amplified from rRP-9 infectious clone was cloned into a lentivirus-expression-vector, pLKO_AS3w-puro (RNAi Consortium, Academia Sinica, Taipei, Taiwan) at restriction sites of *Asc*I and *Pst*I to construct a plasmid named JCprME-pLKO_AS3w-puro. HEK293NT cells were then co-transfected with packaging pCMVΔR8.91, envelope VSV-G pMD.G, and JCprME-pLKO_AS3w-puro to produce lenti-pseudoviral particles. Vero cells were transduced by these pseudoviral particles to integrate *CprME* gene into the genome and then were selected by 10 μg/ml puromycin (Sigma-Aldrich) in MEM supplemented with 10 % FBS and 2 mM L-glutamine with replacement of the medium everyday. Cells were cultured for 2 to 4 weeks and puromycin-resistant colonies were obtained.

### Preparation of JEV SRIPs and titration

To generate SRIPs, JCprME cells were transfected with 400-ng JRP or JRPSP70 using the Lipofectamine 2000 reagent (Invitrogen) according to the manufacturer’s instructions. At day six, the cultured supernatants denote as passage 0 (P0) were harvested and centrifuged to remove cell debris at 1200 rpm for five minutes. SRIPs from P0 were amplified by consecutive passages in JCprME cells at MOI 0.1 in 1X MEM plus 2 % FBS, and SRIPs in the supernatants were often harvested at days seven post-infection and then were further concentrated by hollow fiber tangential flow filtration (100 kDa MACO, 115 cm^2^, modified Polyethersulfone, Spectrum laboratories, Rancho Dominguez, CA).

SRIP or SRIP-SP70 titers were determined by immunoplaque assay. Briefly, CprME cells grown in 6-well plates were infected by the indicated SRIP in a 10-fold serial diluted manner. After 1 h at 37 °C, the infected cells were washed twice with 1X PBS and then overlaid with 1 % agarose (SeaPlaque®, Agarose, Lonza) containing MEM with 2 % FBS. After 4 days incubation, the cells in 6-well plates were fixed by 10 % formaldehyde for 1 h, washed three times with 1X PBS contain 0.1 % tween 20, and permeabilized with 1 % Triton X-100 for 10 min. To view plaques, these cells were stained with anti-JEV NS1 plus alkaline phosphatase-conjugated secondary antibody (Sigma-Aldrich) by adding NBT/BCIP substrate (Promega) to develop the color. SRIPs were titrated manually as plaque forming units per ml (PFU/ml).

### Western blot analysis

Protein samples from infected cells or supernatants were mixed with 1X lysis buffer (proJET® Mammalian Cell lysis reagent, Fermentas) containing 1X protease inhibitor (cOmplete™, Roche). The samples were treated without or with boiling, separated by 10 % SDS-polyacrylamide gel electrophoresis, and transferred to a PVDF membrane (Immobilon®-P, Millipore). The membranes were blocked with 5 % non-fat skim milk in PBST and then reacted with the primary antibodies of JEV specific NS1 (1:1000; J2-54), E (1:1000; J1-85) monoclonal antibodies (MAb), or EV71-VP1 polyclonal antibody (PAb) (1:500; PAB7630-B01P, Abnova). The resulting blots were treated with a horseradish peroxidase-conjugated secondary antibody (1:5000; Jackson ImmunoResearch Laboratories, Inc), and visible signals were developed by enhanced chemiluminescence (ECL, immobilon® Western, Mllipore).

### Indirect immunofluorescence assay (IFA)

To see spreading capability of SRIP, JCprME cells in 6-well plates were infected by 25-PFU of the given SRIPs, and after incubation for the indicated time points, the infected cells were fixed with menthol and acetone (1:1) solution for five minutes at room temperature and then washed twice with PBS plus 5 % skim milk. The primary JEV-specific NS1-MAb, J2-54 (1:1000), in 5 % skim milk/PBS was added for 2 h at room temperature, washed with PBS, and then incubated with FITC-conjugated secondary goat anti-mouse antibodies (1:1000; Jackson Immuno Research Laboratories, Inc) for 1 h at room temperature. Finally, the cells were stained with DAPI (1:1000; Sigma-Aldrich) and imaged by a fluorescence microscope (Nikon Eclipse TE2000- U, Tokyo, Japan).

### Mouse immunization and serum sample collection

Group of 5-week-old ICR female mice (BioLASCO Experimental Animal Center, Taipei, Taiwan) were intra-peritoneally (i.p.) immunized with the negative control PBS, SRIP or SRIP-SP70. For prime-boost immunization protocol, the second dose of the same antigen was given at 2 weeks afterward. Serum samples were collected from facial vein at weeks 2, 4, 6, and 8. The sera were heated at 56 °C for 30 min to inactivate complements and then stored at −80 °C until use. All experiments were performed in the AAALAC-accredited Center of Laboratory Animals at the National Defense Medical Center, Taipei, Taiwan. All animal experiments were carried out according to the guidelines outlined by Council of Agriculture, Executive Yuan, Republic of China. The animal protocol was approved by the Institutional Animal Care and Use Committee (IACUC) of the National Defense Medical Center with permit numbers IACUC-14-118 and IACUC-14-254.

### Serum specific anti-SP70 IgG

The total anti-SP70 IgG and their subtypes were examined by an enzyme-linked immunosorbent assay (ELISA). Briefly, 96-well ELISA plates (Costar) were coated with 10 μg/ml of synthetic SP70 peptide (purchased from NIIDV peptide synthesis core facility in Taiwan) as antigen for overnight at 4 °C. Plates were washed once with PBST (0.05 % Tween 20 in PBS) and then blocked with 5 % non-fat skim milk/PBS for 2 h. After incubation, plates were washed twice with PBST. The two-fold serially diluted sera (2^7^ to 2^15^) in 1 % BSA/PBS were added, and after incubation for 2 h and six times of washes, the secondary antibody was added, in which horseradish-peroxidase (HRP)-conjugated goat anti-mouse IgG (1:5000 dilution, Cappel Laboratories, Malvern, PA) was for total IgG titration and affinity purified HRP-conjugated rabbit anti-mouse was for IgG1, IgG2a, or IgG2b (1:2000 dilution, ZYMED® Laboratories, South San Francisco, CA). Plates were washed eight times with PBST and color was developed by adding 3,3′, 5,5′- tetramethylbenzidine (TMB) substrate (KPL) for 20 min, stopped with 1 N H_2_SO_4_, and detected at an optical density (OD) of 450 nm by ELISA plate reader. For the total anti-SP70 IgG and subtypes IgG titration, the positive OD value was obtained by cutting off 1.5 times of OD values from group of PBS-immunized serum.

### Lethal challenge

Because EV71 infection caused no apparent clinical symptoms in adult ICR mice, one-day-old ICR mice were used for lethal challenge using EV71 MP4. Groups of 5-week-old female ICR mice were immunized with PBS, SRIP or SRIP-SP70 and for prime-boost protocol the mice were received the second immunization of the same antigens 2 weeks afterward. Mice were allowed to mate at the day when the final immunization was completed. Within 24 h, the one-day-old neonatal mice were challenged i.p. with EV71/MP4 (4 LD_50_) or JEV rRP-9 (1 × 10^3^ PFU) virus in 50 μl/mouse. The survival rates were monitored daily for 14 days.

### Statistical analysis

The data were presented as the mean ± the standard error. Statistical significance was determined using GraphPad Prism 6.0 software (GraphPad Software, San Diego, CA) for data analysis. In vitro data from cell-based experiments were analyzed by unpaired *t* test. *p* < 0.05 was considered significant.

## Results

### Construction of JEV-based replicon expressing EV71 neutralizing epitope SP70 and establishment of replicon packaging cells JEV-CprME

**Fig. 1:**
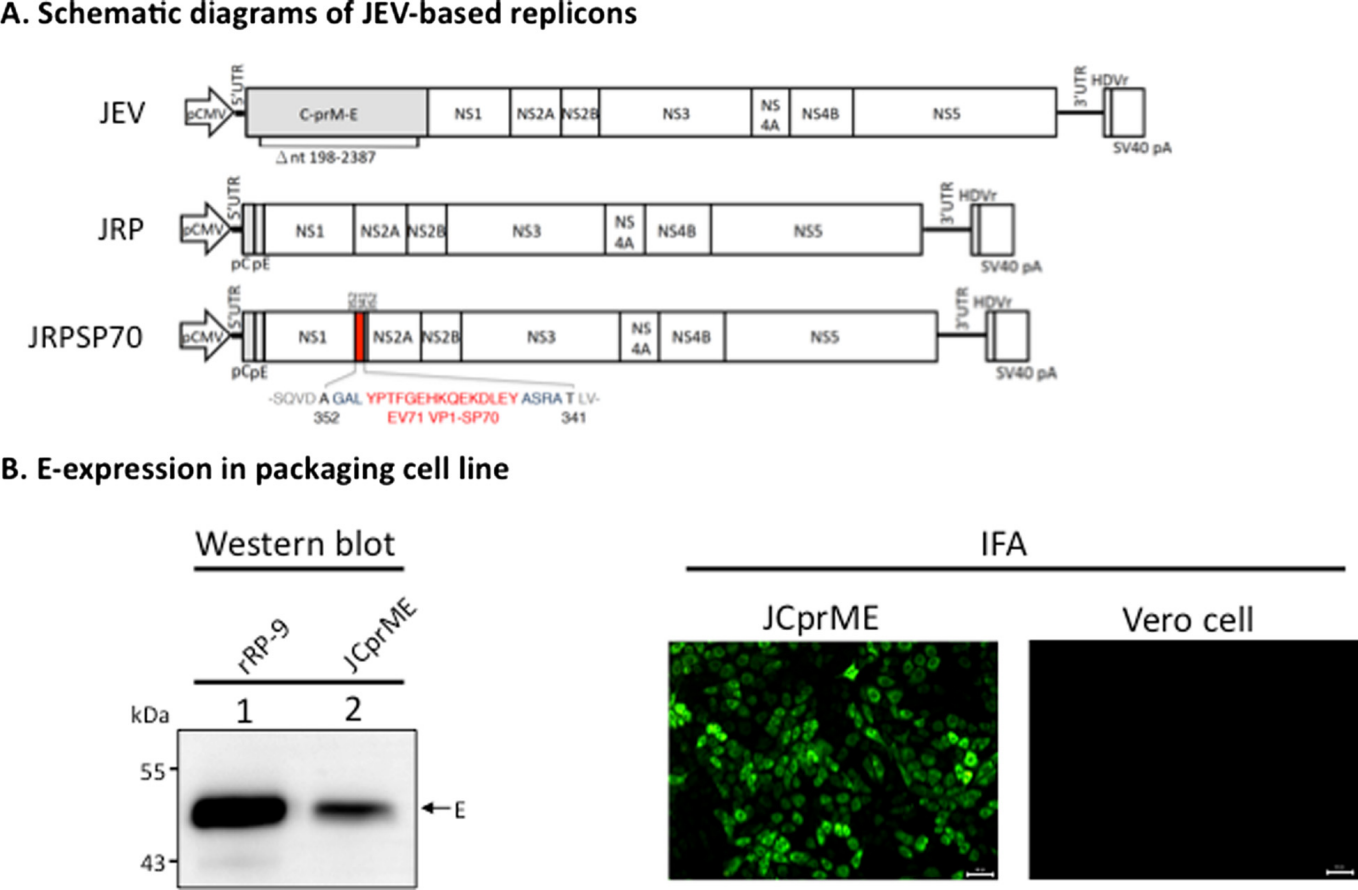
JEV-based replicons and JEV-CprME packaging cells. **a** Schematic diagrams of JEV-based replicons. JEV infectious cDNA driven by CMV promoter encodes three structural (*gray box*) and seven nonstructural (*open boxes*) proteins. JRP was derived from JEV infectious cDNA by deletion of nucleotides from 198–2387, where most of structural proteins were deleted. JRPSP70 included a SP70 epitope (red box) that was fused at C-terminal of NS1. **b** The packaging cells, named JCprME, were created by using pseudo-lentiviral particles containing structural genes *CprME* to infect Vero cells and were selected by puromycin. The E-expression of JCprMC cells was determined by Western blot (*left panel*) and IFA (*right panel*) using MAb against JEV E protein, and Vero cells served as a negative control. Samples from rRP-9-infected lysates serve as a positive control for protein position of E. The arrow denotes the position of E proteins. Scale bar is 50 μm in length

Starting from our previous replicons J-R2A [17] and J-R2ASP70, from which the genes of Renilla luciferase (RLuc) and 2A-protease were deleted and the new replicons of JRP and JRPSP70, respectively, resulted (Fig. 1a). To package replicon RNA genomes into SRIPs, we engineered Vero cells to stably express JEV CprME proteins by lentovirual system, and after puromycin selection for a proper time period, the stable cells, named JCprME, were constructed, which could accurately express JEV-E proteins as evidenced by Western blotting and immunofluorescent assay (Fig. 1b).

### Generation of SRIPs from JEV-based replicons could replicate and spread within packaging cells JCprME

**Fig. 2:**
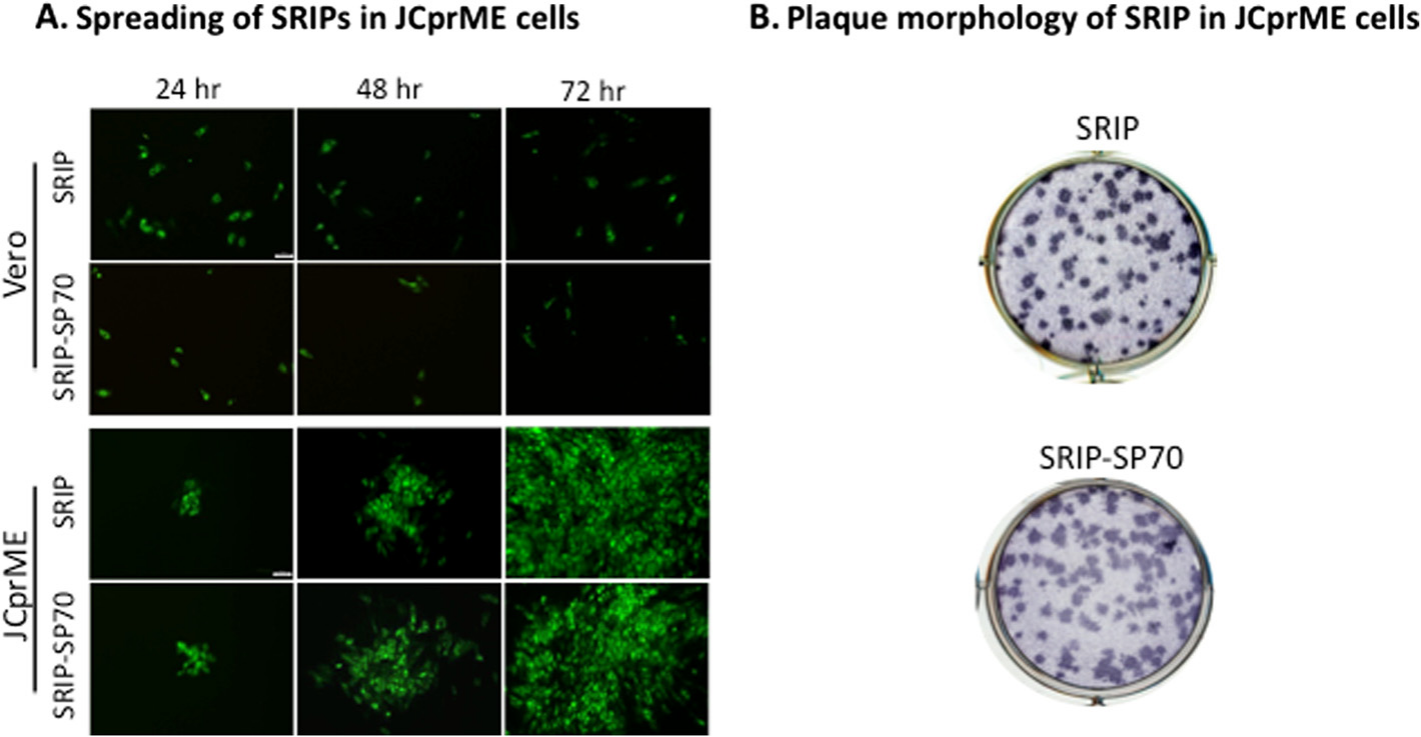
Packaging, spreading and plaque morphology of SRIP. **a** Spreading of SRIPs in JCprME cells. The Vero cells (*upper panel*) or JCprME cells (lower panel) were seeded in 6-well plates to reach a confluent monolayer, which were inoculated with SRIP or SRIP-SP70 for the indicated time periods. The resulting cells were fixed for IFA using MAb specific for JEV NS1. **b** Plaque morphology of SRIP and SRIP-SP70 were examined at day 4 post-infection by immunoplaque assay using MAb specific for JEV NS1. Scale bar is 50 μm in length

To generate pseudo-type virus particles, we transfected JCprME cells with plasmid containing JRP or JRPSP70 replicon, and supernatants from the transfected cells were harvested on days 6 post-transfection for collection of SRIP and SRIP-SP70. We next infected Vero or JCprME cells with SRIP or SRIP-SP70 to see how these pseudo-type virus particles replicated and dispersed in cultured cells. Based on immunofluorescence analysis using anti-JEV NS1 MAb, we found that SRIP or SRIP-SP70 could only replicate once but without scattering around in Vero cells with time (Fig. 2a, upper panel). In contrast, both SRIP and SRIP-SP70 could replicate and disperse in JCprME cells as time went by (Fig. 2a, lower panel). Similar plaque morphology of SRIP and SRIP-SP70 could be seen when grown on JCprME cells by immunoplaque assay (Fig. 2b). These results clearly demonstrate that the pseudo-type SRIP and SRIP-SP70 can indeed only replicate once in normal cells.

### Normal multimeric NS1-fusion proteins could be produced from SRIP-SP70 infected JCprME cells

**Fig. 3:**
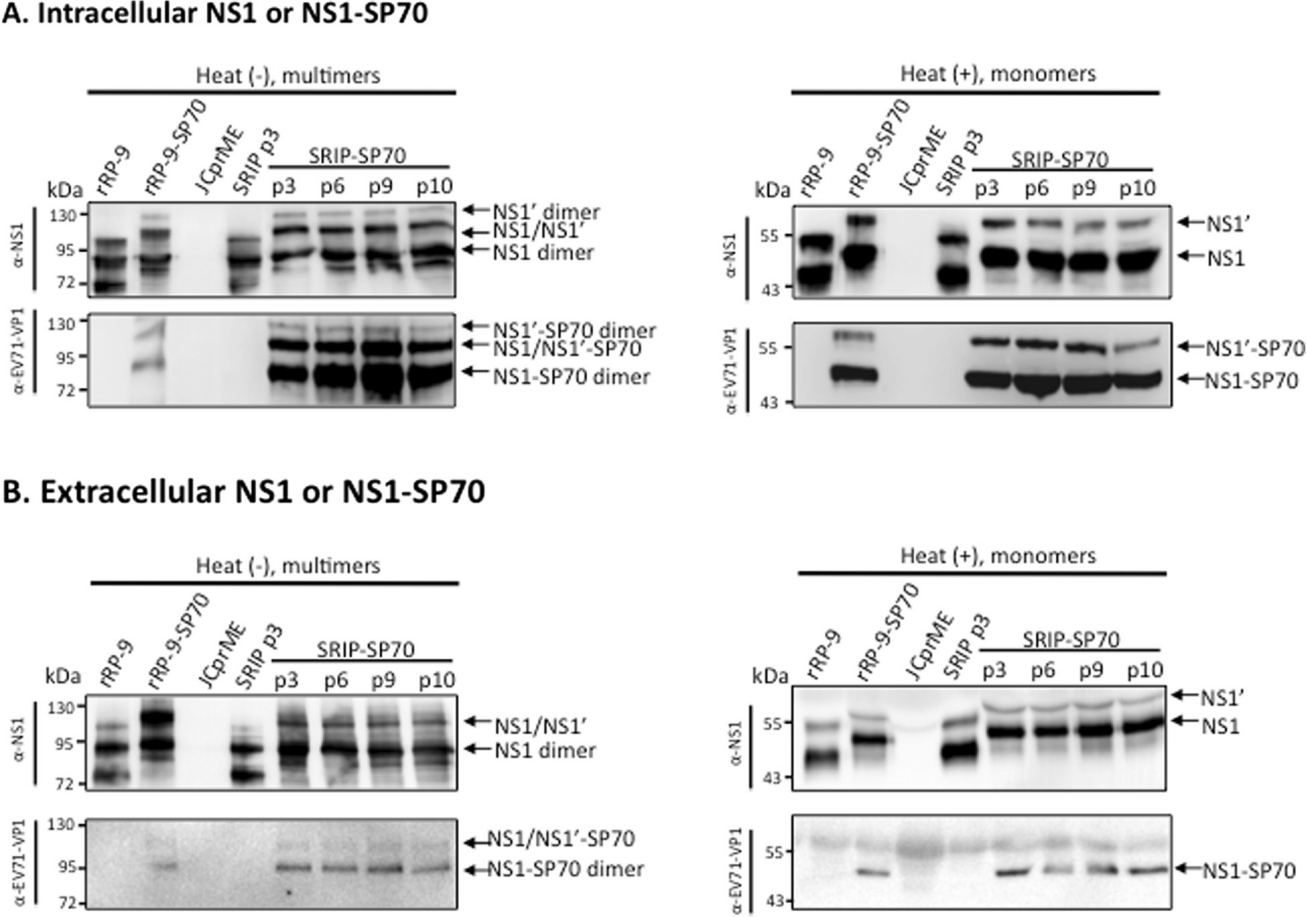
Multimeric NS1 fusion proteins derived from SRIP-SP70 infection. JCprME cells were repeatedly infected by SRIP-SP70 as indicated, and cell lysates were prepared for detection of the intracellular NS fusion proteins (**a**) and supernatants were collected for detection of the extracellular NS fusion proteins (**b**). Analyzed by Western blotting using NS1-specific or SP70-specific antibodies, the protein samples from cell lyastes or supernatants were left unboiled to detect multimeric NS1 fusion proteins (the left parts of panels **a** and **b**), or being boiled to examine monomers of NS1 proteins (the right parts of panels **a** and **b**). Samples from virus infection by rRP-9 or rRP9-SP70 serve as a positive control. Samples from JCprME were mock-infected controls. The arrow denotes the dimer or monomer position of NS1 or NS1-SP70

Since flavivirus NS1 is highly immunogenic to the host due to its multimeric formation [5], we examined the effects of EV71 epitope insertion on dimerization and secretion of NS1-SP70 fusion proteins during infection. We first infected JCprME cells with SRIP-SP70 in multiple-passage manner, and the viral antigens in cell lysates or culture medium were collected for western blotting using antibodies specifically recognizing JEV-NS1 or EV71-VP1. These protein samples were prepared through either being boiled to detect monomeric or leaving unboiled to detect dimeric NS1-EV71-epitope fusion proteins. NS1’, a longer version of NS1 was the result from a ribosomal frame-shifting mechanism, is only expressed by JE serocomplex flaviviruses including JEV [18]. As compared with the parental virus rRP-9 shown in Fig. 3, all NS1-SP70 fusion proteins from the indicated SRIP-SP70 also had a longer NS1’ version, could properly dimerize in both intracellular and extracellular unheated samples (Fig. 3, the left parts of panels a and b), and were readily secreted from infected cells (panel B). Importantly, we found that all SRIPs could secrete soluble NS1 and NS1’ in their correct multimeric forms as their parental rRP-9, from infected cells (Fig. 3). Together, these results clearly illustrate that fusion of heterologous epitopes with JEV-NS1 does not impair the abilities of SRIP-SP70 in terms of NS1’ processing, NS1 dimerization and secretion.

### SRIP-SP70 could confer a dual immunity against JEV or EV71 lethal challenges

**Fig. 4:**
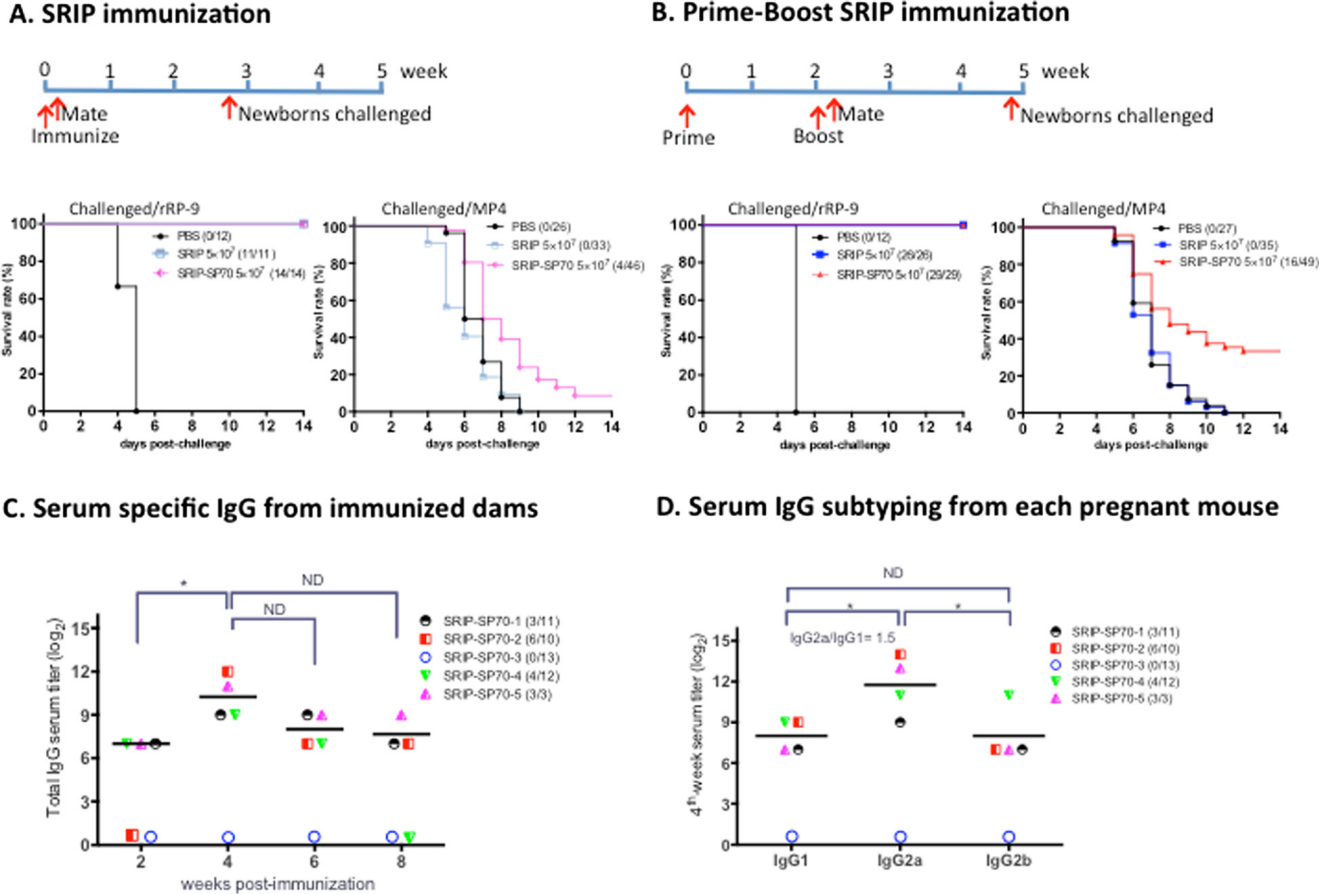
SRIP-SP70 confers dual protective immunity against JEV or EV71 challenge via Th1-predominant immune response. Survival rates were evaluated for groups of one-day-old ICR newborns from their mothers that had been immunized with PBS alone, SRIP or SRIP-SP70 using SRIP immunization protocol (**a**) or by prime-boost SRIP immunization protocol (**b**). These newborns were lethally challenged by either 1 × 10^3^ PFU of JEV rRP-9 (the left part of panels **a** and **b**) or 4 LD_50_ of EV71/MP4 (the right part of panels **a** and **b**). **c** Serum specific IgG from immunized dams. To measure serum specific anti-SP70 IgG titers by ELISA, the SRIP-SP70-immunized sera were collected from groups of female ICR mice at the indicated times after prime-boost SRIP immunization protocol. **d** Serum IgG subtyping from each pregnant mouse. The specific anti-SP70 IgG in serum was subtyped by ELISA isotype binding assays, and each individual serum was collected and analyzed 4 week after immunization protocol completed in (**c**). Data are shown as mean for group mice (*n* = 5). The symbols represent immunized ICR mice and parentheses indicate survival rate of newborns after challenges. The serum titer that was less than 2^7^ dilutions will be marked as 0. ELISA plates were coated with 10 μg/ml of synthetic SP70 peptide as antigen. Star * indicates p value < 0.05. ND means no difference

To examine whether immunization of SRIP or SRIP-SP70 could protect mice from homologous JEV lethal challenge, we inoculated 1 × 10^3^ PFU of JEV rRP-9 to groups of one-day-old newborn mice borne to their immunized mothers. We found that all the newborns from dams that were immunized with 5 × 10^7^ PFU of SRIP or SRIP-SP70 could survive, whereas all the newborns from PBS-immunized dams were killed within 6 days after the lethal challenge of JEV rRP-9 (Fig. 4, the left part of panels a and b). It remained 100 % protective when the immunized dose was reduced to 1 × 10^5^ PFU of SRIP or SRIP-SP70 (data not shown).

As EV71 infection caused no apparent clinical symptoms in adult ICR mice [19], we opted to intra-peritoneally challenge one-day-old ICR mice by a mouse-brain adapted strain EV71/MP4, whose 50 % lethal dose (LD_50_) was approximately 32 PFU and the minimum lethal dose to kill all infected newborns was 4 LD_50_. To investigate whether SRIP-SP70 could also protect the immunized mice from heterologous EV71 lethal challenge, again, we intra-peritoneally injected a lethal dose of EV71/MP4 to groups of one-day-old ICR newborns from their mothers immunized with PBS alone, or 5 × 10^7^ PFU of either SRIP-SP70 or SRIP as a control. We found that, compared with controls of PBS or SRIP immunization, when we used the SRIP immunization protocol (Fig. 4a), only 8 % (4/46) of the challenged newborns survived; in contrast, when using the prime-boost SRIP immunization protocol (Fig. 4b), we observed that 32 % (16/49) of the challenged newborns survived.

To understand the specific anti-SP70 antibody responses, we examined each immunized serum collected at different time points by ELISA from dams using prime-boost SRIP immunization protocol. As the results shown in Fig. 4c, the specific serum titers from most of the pregnant mice tested could reach the plateau 4 weeks after immunization protocol completed, at the period roughly one week before giving birth. Since the serum levels of IgG2a and IgG1 are often used to define how immunity undergoes the Th1- or Th2-predonminant responses when the hosts are under infections, and the IgG2a/IgG1 ratios are used to predict which T-cell phenotype can be induced by a given vaccination [20–22]. We therefore in Fig. 4d examined levels of the indicated subserotypes of serum specific derived from mice at week 4 in Fig. 4c. We found that IgG2a levels from immunized dams were significant higher than those of IgG1 or IgG2b, and almost all IgG2a/IgG1 ratios were >1, indicating a Th1-biased immune response (Fig. 4d). Interestingly, we identified a non-responder, named SRIP-SP70-3, in Fig. 4c and d, whose serum specific IgG levels were as low as the background. Pregnant SRIP-SP70-3 subsequently had given birth to 13 newborns that were included in the challenge experiment shown in Fig. 4b.

## Discussion

We in this study used JEV replicon-based platform to generate SRIPs as a vaccine, which contains JEV subgenomic RNA wrapped by virus structural proteins derived *in trans* from stable packaging JCprME cells to form a typical pseudo virus particles. However, replicon-based SRIP and SRIP-SP70 cannot be packaged and spread out in Vero cells because these cells did not express CprME structure proteins that are required for forming infectious particles. SRIPs can only replicate within their first infected cells but not in the surrounding cells due to lack of CpME proteins provided *in trans* by Vero cells. Starting from using replicon-containing plasmid DNA, we first obtained small amounts of SRIPs from the transfected JCprME cells and subsequently could attain sufficient amounts of SRIPs through repeat infections in JCprME cells. With a proper optimization, this process appears to be the reliable and streamline protocol for flaviviral RNA vaccine production.

Using the prime-boost SRIP immunization protocol (Fig. 4b) but not the SRIP immunization (once) protocol (Fig. 4a), we found that 32 % (16/49) of the newborns from their mothers immunized with SRIP-SP70 could survive from the subsequent lethal EV71 challenge. Since the control groups of PBS or SRIP immunization in the above experiment (Fig. 4b) showed no protective capability, we therefore conclude that the backbone of JEV replicon plays no role in the protection against EV71 resulting from SRIP-SP70 immunization. As a result, it is the neutralizing epitope SP70 fused with NS1 protein that plays a critical role in providing such a protective immunity from SRIP-SP70 against heterologous EV71 lethal challenge. On the other hand, we clearly illustrated that the backbone of JEV replicons used in this study could readily confer a complete protection from homologous JEV lethal challenge (Fig. 4a and b). Our findings thus provide a proof of concept about that SRIP-SP70, produced by JEV replicon-based platform established from this study, confers a dual protective immunity against subsequent lethal challenges from JEV as well as EV71 infections.

Interestingly, we later discovered that one of the SRIP-SP70-3-immunized dams in fact appeared to be a non-responder in terms of its extremely low serum specific IgG responses (Fig. 4c and d), and this mouse gave birth to 13 newborns that had already been included in the abovementioned lethally EV71-challenged experiment (Fig. 4b). These newborns were all killed by subsequent EV71 lethal challenge. Since ICR mice are genetically outbred, we therefore consider that there should be 44 % (16/36) of the challenged newborns survived among the responders when using prime-boost SRIP-SP70 immunization protocol. These observations seem to suggest a positive correlation between the titers of serum specific IgG in SRIP-SP70-immnunized mother dams and the survival rates of their newborns to face EV71 challenge. An insufficient EV71-specific Th1 response has been implied to be associated with immunopathogenesis of EV71-related pulmonary edema [23]. A strong cellular immunity especially biased toward Th1 response through vaccination is thought to be an important strategy for defeating EV71 infections. We in this study showed SRIP-SP70 could trigger Th1-biased immune response (Fig. 4d), which may thus play a protective role in newborn mice when they were lethally challenged by EV71. Although SP70 peptide is highly conserved amongst many EV71 strains and the antisera raised against SP70 were able to neutralize many different EV71 strains [13], other neutralizing epitopes identified, such as VP2-28 [22], should be included in our platform to improve its EV71 protection capability. However, in addition to MP4, different EV71 strains are needed to be further tested for the protective efficacy of SRIP-SP70 immunization in the future.

## Conclusions

Here we established JEV replicon-based platform to generate SRIP vaccine that expressed heterologous EV71 neutralizing epitope. Such pseudo-infectious virus particles, although are not genuine viruses, can closely mimic live virus infection to elicit immune responses within one round of viral life cycle. We found that, in addition to thwart JEV lethal challenge, the female outbred ICR mice immunized with JRPSP70 SRIPs by prime-boost protocol could also provide their newborns certain degree of protection against subsequent EV71 lethal challenge. These results therefore clearly demonstrate that this vaccination strategy indeed can confer immunized hosts a dual protective immunity against subsequent lethal challenge from JEV or EV71.
